# Supplementary material for: Association between Adverse Childhood Experiences and Multiple Sclerosis in Icelandic Women—A Population-Based Cohort Study
Source: Brain Sci. 2022 Nov 16;12(11):1559. doi: 10.3390/brainsci12111559 (PMC9688793; doi:10.3390/brainsci12111559)
Supplement: Supplementary file 1 [file brainsci-12-01559-s001.zip › brainsci-1975525-supplementary.pdf]

Table S1. ACE-IQ Categories and Questions with Frequency Scoring

Category of ACE	Questions	Required Minimum Frequency
**Abuse**		
Physical	Did a parent, guardian or other household member spank, slap, kick, punch or beat you up?	Many times
	Did a parent, guardian or other household member hit or cut you with an object, such as a stick (or cane), bottle, club, knife, whip etc?	
Emotional	Did a parent, guardian or other household member yell, scream or swear at you, insult or humiliate you?	Many times
	Did a parent, guardian or other household member threaten to, or actually, abandon you or throw you out of the house?	
Sexual	Did someone touch or fondle you in a sexual way when you did not want them to?	Ever
	Did someone make you touch their body in a sexual way when you did not want them to?	
	Did someone attempt oral, anal, or vaginal intercourse with you when you did not want them to?	
	Did someone actually have oral, anal, or vaginal intercourse with you when you did not want them to?	
**Neglect**		
Physical	Did your parents/guardians not give you enough food even when they could easily have done so?	Many times
	Were your parents/guardians too drunk or intoxicated by drugs to take care of you?	
	Did your parents/guardians not send you to school even when it was available?	
Emotional	Did your parents/guardians understand your problems and worries?	Rarely or never
	Did your parents/guardians really know what you were doing with your free time when you were not at school or work?	Rarely or never really
**Household dysfunction**		
Family violence	Did you see or hear a parent or household member in your home being yelled at, screamed at, sworn at, insulted or humiliated?	Many times
	Did you see or hear a parent or household member in your home being slapped, kicked, punched or beaten up?	A few times or many times
	Did you see or hear a parent or household member in your home being hit or cut with an object, such as a stick (or cane), bottle, club, knife, whip etc.?	A few times or many times
Parental separation or divorce	Were your parents ever separated or divorced?	Yes
	Did your mother, father or guardian die?	
Substance abuse	Did you live with a household member who was a problem drinker or alcoholic, or misused street or prescription drugs?	Yes

Incarcerated household member	Did you live with a household member who was ever sent to jail or prison?	Yes
Mental illness	Did you live with a household member who was depressed, mentally ill or suicidal?	Yes
**Violence**		
Community violence	Did you see or hear someone being beaten up in real life?	Many times
	Did you see or hear someone being stabbed or shot in real life?	
	Did you see or hear someone being threatened with a knife or gun in real life?	
Bullying	How often were you bullied?	Many times
Collective violence	During the first 18 years of your life, were you exposed to war/collective violence (e.g. from gangs or police)?[a]	Yes/No
	Were you forced to go and live in another place due to any of these events?	Ever
	Did you experience the deliberate destruction of your home due to any of these events?	
	Were you beaten up by soldiers, police, militia, or gangs?	
	Was a family member or friend killed or beaten up by soldiers, police, militia, or gangs?	

[a] screening question; participants that responded affirmatively received four follow-up questions
